# LONGITUDINAL EFFECTS OF HYPERTENSION ON COGNITIVE PERFORMANCE IN OLDER ADULTS

**DOI:** 10.1093/geroni/igac059.2250

**Published:** 2022-12-20

**Authors:** Allison Moll, John L Woodard

**Affiliations:** Wayne State University, Detroit, Michigan, United States; Wayne State University, Detroit, Michigan, United States

## Abstract

Hypertension impacts many older adults, but it is still not clear whether it has a negative effect on cognitive performance in older adults. The purpose of this study was to assess the longitudinal effect of hypertension on cognition in older adults (Mage=75.6 years, SD=8.3). Participants came from the National Alzheimer’s Coordinating Center database. The cognitive assessment included the MoCA, Digit Span, Trail-Making Test A and B, WAIS-R Digit Symbol, Category Fluency, and Letter Fluency. Linear mixed effects modeling examined the random and fixed effects of clinician-assessed hypertension, months since first study visit, sex, age, and the interaction between hypertension and time since first visit on cognitive performance across five annual study visits. Results showed that hypertension had a significant main effect on Category Fluency, Trails B, Letter Fluency, and Digit Span–Forward and Backward. However, effect sizes were quite small (ηp2 range: 3.93x10-4 – 1.73x10-3). Main effects of age and months since first visit were significant predictors of all cognitive measures, such that older age was associated with worse cognitive performance and more months since the first visit was associated with better cognitive performance. This positive association is perhaps suggestive of practice effects across study visits. A significant interaction between hypertension and months since first visit for Category Fluency and Trails B showed that hypertensives and non-hypertensives performed differently at the initial visit but similarly by the last visit. However, effect sizes were small (ηp2 range: 3.86–9.64x10-4). These results suggest hypertension effects on cognition in older adults are minimal.

